# Clinical profile of ocular allergy and its impact on quality of life in Brazilian children and adolescents

**DOI:** 10.1590/1984-0462/2025/43/2025116

**Published:** 2025-11-14

**Authors:** Luiza Moulin Marino, Herberto José Chong, Cristine Secco Rosario, Layra Layane de Andrade Belo Rebouças, Ana Caroline Dela Bianca Melo, Dirceu Solé, Gustavo Falbo Wandalsen

**Affiliations:** a Universidade Federal de São Paulo, São Paulo, SP, Brazil.; b Universidade Federal do Paraná, Curitiba, PR, Brazil.; c Universidade Federal de Pernambuco, Recife, PE, Brazil.

**Keywords:** Allergic conjunctivitis, Keratoconjunctivitis, Vernal keratoconjunctivitis, Atopic conjunctivitis, Conjuntivite alérgica, Ceratoconjuntivite, Ceratoconjuntivite primaveril, Conjuntivite atópica

## Abstract

**Objective:**

To describe the symptoms, severity, and impact on quality of life of ocular allergy in children and adolescents followed up in referral centers in Brazil.

**Methods:**

Cross-sectional, observational study carried out in three referral centers for pediatric allergy in children and adolescents (5–17 years) diagnosed with ocular allergy. Clinical data was obtained from the medical records. Patients scored the intensity of their ocular and nasal symptoms using a visual analog scale. The impact of ocular allergy on their quality of life was assessed using a specific questionnaire (*Quality of Life in Children with Keratoconjunctivitis* — QUICK) and the EQ-5D visual analog scale.

**Results:**

196 participants were included, 37.8% with vernal or atopic keratoconjunctivitis and 62.2% with seasonal or perennial allergic conjunctivitis. The most common allergic comorbidity was rhinitis. The most intense symptoms in both groups were pruritus and ocular hyperemia. The main trigger for allergic symptoms, according to patients’ perceptions, was house dust. Participants with keratoconjunctivitis had a significantly lower median score on the EQ-5D than those with seasonal/perennial allergic conjunctivitis. There was a higher prevalence of medication use across all the classes investigated among patients with keratoconjunctivitis. Ocular lubricants were underused by patients, especially in the milder forms of the disease.

**Conclusions:**

The clinical presentation of ocular allergy is heterogeneous, with pruritus and hyperemia being the most common symptoms. House dust was reported as the main trigger of symptoms. Children and adolescents with keratoconjunctivitis experience higher impact on quality of life during symptomatic periods than those with seasonal and perennial allergic conjunctivitis.

## RESUMO

**Objetivo:**

Descrever os sintomas, a gravidade e o impacto da alergia ocular na qualidade de vida de crianças e adolescentes acompanhados em centros de referência no Brasil.

**Métodos:**

Estudo transversal e observacional realizado em três centros de referência em alergia pediátrica com crianças e adolescentes (5–17 anos) diagnosticados com alergia ocular. Dados clínicos foram obtidos dos prontuários médicos. Os pacientes classificaram a intensidade dos sintomas oculares e nasais por meio de escala visual analógica. O impacto da alergia ocular em sua qualidade de vida foi avaliado por meio de questionário específico (Quality of Life in Children with Keratoconjunctivitis — QUICK) e da escala visual analógica EQ-5D.

**Resultados:**

Foram incluídos 196 participantes, 37,8% com ceratoconjuntivite vernal ou atópica e 62,2% com conjuntivite alérgica sazonal ou perene. A comorbidade alérgica mais comum foi a rinite. Os sintomas mais intensos em ambos os grupos foram prurido e hiperemia ocular. O principal desencadeante dos sintomas alérgicos, segundo a percepção dos pacientes, foi a poeira doméstica. Participantes com ceratoconjuntivite apresentaram pontuação significativamente menor no EQ-5D do que aqueles com conjuntivite alérgica sazonal/perene. Houve maior prevalência do uso de medicamentos de todas as classes investigadas entre os pacientes com ceratoconjuntivite. Os lubrificantes oculares foram subutilizados pelos pacientes, especialmente nas formas mais leves da doença.

**Conclusões:**

A apresentação clínica da alergia ocular é heterogênea, sendo o prurido e a hiperemia os sintomas mais comuns. Poeira doméstica foi o desencadeante de sintomas mais relatado. Crianças e adolescentes com ceratoconjuntivite apresentam maior impacto na qualidade de vida durante os períodos sintomáticos do que as com conjuntivite alérgica sazonal/perene.

## INTRODUCTION

 Ocular allergy (OA) is a highly prevalent condition worldwide and comprises a heterogeneous group of inflammatory diseases affecting the conjunctiva, eyelids and cornea, with type I and IV hypersensitivity reactions as the pathophysiological mechanisms.^1^ IgE-mediated OA can be classified into different types, such as seasonal allergic conjunctivitis (SAC), perennial allergic conjunctivitis (PAC), vernal keratoconjunctivitis (VKC) and atopic keratoconjunctivitis (AKC).^1,2^ The symptoms common to all forms of conjunctivitis include ocular or periocular itching, tearing and conjunctival hyperemia. 

 Among the different forms of OA, there is a significant involvement of allergic inflammation in PAC and SAC, where allergic sensitization is observed in around 90% of patients. The etiopathogenesis of keratoconjunctivitis (KC) is more complex, involving Th1 and Th2 inflammatory mechanisms.^2^ VKC and AKC are more severe and less frequent forms of OA, with no national data on their prevalence. Patients with KC have a higher risk of visual loss over time, often associated with anterior segment changes such as corneal opacities, keratoconus and cataracts.^3^

 Global estimates indicate that 10 to 20% of the population is affected by some form of OA, with its impact extending beyond ocular health, significantly diminishing quality of life and reducing productivity for those affected.^1^ These important parameters should always be assessed in the follow-up of patients with chronic conditions. 

 There is a lack of national data on the prevalence of OA, with more consistent information available only on allergic rhinoconjunctivitis. According to the Brazilian results of phase 3 of the *International Study of Allergy and Asthma in Childhood* (ISAAC) study, involving schoolchildren and adolescents, the average prevalence of rhinoconjunctivitis symptoms was 13.3% among schoolchildren and 14.6% among adolescentes.^4^ Specifically assessing the presence of eye symptoms, Geraldini et al. found a prevalence of 20.7% of allergic conjunctivitis among adolescents in the city of Curitiba (PR), with more than half of the adolescents reporting itchy eyes.^5^

 Given this scenario, describing the symptoms and severity of OA in children and adolescents followed up in reference centers in Brazil, as well as the frequency of association with other allergic diseases and the impact of OA on the quality of life of these patients, is of fundamental importance to better understand the disease. 

## METHOD

 A cross-sectional, multicenter, observational study was carried out in three reference centers for pediatric allergy in Brazil, with the Universidade Federal de São Paulo (UNIFESP) as the coordinating center and the others being the Universidade Federal de Pernambuco (UFPE) and the Universidade Federal do Paraná (UFPR). Clinical data was collected between July 2022 and October 2023. 

 We investigated children and adolescents aged between five and 17, an age range defined to ensure the ability to provide valid responses to the instruments applied in the study. All participants had a previous diagnosis of SAC, PAC, VKC or AKC established by an allergist and/or ophthalmologist based on clinical criteria (symptoms, medical history and ophthalmologic examination), with a minimum disease duration of six months, as verified through a retrospective review of medical records.^6^ Individuals with incomplete clinical data were not included in the analysis. 

 Based on the estimate of KC cases eligible for admission to the study and being followed up at the three participating centers, the inclusion of 180 children and adolescents with OA was planned, two-thirds (n=120) with SAC or PAC and one-third (n=60) with VKC or AKC. Patients diagnosed with SAC or PAC were grouped for analysis, as were those diagnosed with VKC or AKC. The groups were matched for age (±1 year) across study sites. 

 Clinical data was obtained from the medical records and supplemented by interviews with the patients and/or their families, and by them filling out a clinical form drawn up for the study, containing the following information: personal details; age at onset of ocular symptoms and diagnosis of ocular allergy; frequency of ocular symptoms; apparent triggers of allergic crises; concomitance of other allergic diseases; current treatments; previous ocular surgeries. 

 The design of the trigger questionnaire aimed to assess, from the patients’ perspective, the main factors triggering their ocular symptoms, including not only allergens but also various irritants. Patients were asked whether they noticed the onset of ocular symptoms following exposure to house dust, dogs, cats, pollens, mold, light/brightness, temperature changes, wind, pollution, and the presence of respiratory infections. 

 Patients scored the intensity of their ocular symptoms (itching, tearing, hyperemia, photophobia and pain) and nasal symptoms (itching, runny nose, nasal obstruction and sneezing), based on the two weeks before entering the study, using a visual analog scale ranging from 0 to 10, adapted from the visual analog scale validated for allergic rhinitis.^7^

 The impact of ocular allergy on patients’ quality of life was assessed using the *Quality of Life in Children with Keratoconjunctivitis* (QUICK) questionnaire, which was answered by the children and adolescents themselves, with the help of their guardians when necessary.^8^ The QUICK questionnaire, validated for Brazilian culture, consists of 16 items divided into two domains: related symptoms and limitations of daily activities. All the items evaluated, referring to the last two weeks, are scored according to frequency of occurrence (1=never, 2=sometimes, and 3=always) and the sum of all 16 items allows the total score to be calculated (16 to 48 points), with quality of life being worse the higher the score.^8,9^

 Quality of life was also measured using the EQ-5D visual analog scale, in which children and adolescents give a score (0 to 100) for their health in asymptomatic moments and during OA exacerbations, with the score being worse the closer it is to 0.^10^

 Categorical variables were expressed by their absolute and relative frequencies, and their possible differences were estimated using Pearson’s chi-square test with Yates’ correction. Two groups were compared regarding quantitative variables using the Student’s t-test for independent symmetrical continuous variables, two-tailed hypothesis, while the non-parametric Mann-Whitney test was used to compare asymmetrical continuous variables. A minimum significance level of 5 and a 95% confidence interval (95%CI) were considered for all tests. The software used was IBM Statistical Package for the Social Sciences (SPSS) Statistics® v29.0.1.0. 

 The study began after approval by the Human Research Ethics Committees of all the participating centers. Signatures on the Free and Informed Consent Form and the Free and Informed Assent Form were obtained from all parents and/or guardians and the study participants. 

## RESULTS

 Of the 197 participants initially included in the study, one was not considered for analysis due to insufficient clinical data. The final number of participants was 196; 74 (37.8%) were diagnosed with VKC or AKC and 122 (62.2%) with SAC or PAC. Ninety-six patients were from São Paulo (UNIFESP), 66 from Paraná (UFPR) and 34 from Pernambuco (UFPE). The median age at the time of inclusion in the study was 12 years (interquartile range — IQR 9–14) and 61.2% of the patients were male. The most common allergic comorbidity was rhinitis (91.8%), followed by asthma (57.1%), atopic dermatitis (55.1%) and food allergies (18.4%). Table 1 shows the distribution of gender, age and comorbidities according to the diagnosis of ocular allergy. 

**Table 1:** Clinical and demographic data of children and adolescents with ocular allergy.

		VKC/AKC (n=74)	SAC/PAC (n=122)	p-value
Male sex (%)		50 (67.5)	70 (57.3)	0.156
Current age (years)		12 (10–14)	11 (9–14)	0.073
Age at onset of ocular symptoms (years)		4 (2–6)	3,5 (2–6)	0.939
Age at diagnosis of ocular allergy (years)		7 (4–10)	6 (4–9)	0.404
Allergic comorbidities (%)				
	Allergic rhinitis	69 (93.2)	110 (90.1)	0.283
	Asthma	40 (54.0)	72 (59.0)	0.496
	Atopic dermatitis	43 (58.1)	65 (53.2)	0.510
	Food allergy	16 (21.6)	20 (16.4)	0.359

Legend: VKC:vernal keratoconjunctivitis; AKC: atopic keratoconjunctivitis; SAC: seasonal allergic conjunctivitis; PAC: perennial allergic conjunctivitis. Data are expressed as number (%) or median (interquartile range).

**Figure 1:**
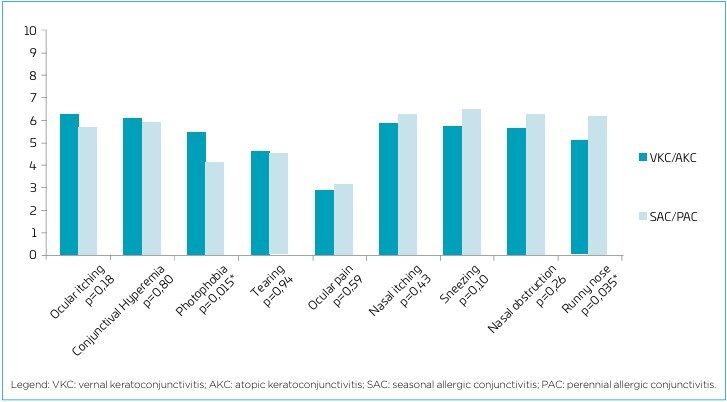
Mean intensity values of ocular and nasal symptoms over the past two weeks in patients with vernal keratoconjunctivitis and atopic keratoconjunctivitis and those with seasonal allergic conjunctivitis and perennial allergic conjunctivitis (n=187), assessed using a 0–10 visual analog scale.

 Regarding ocular symptom scores, assessed by the patients using the visual analog scale, the most intense symptoms in both groups were pruritus and ocular hyperemia, both with a mean above 5 (Figure 1). Photophobia was the only symptom with significantly different values between the groups, being more intense in the group diagnosed with KC. 

 For nasal symptoms score, there was a significant difference between the groups only concerning the occurrence of coryza, which was more intense in patients with SAC or PAC, as shown in Figure 1. 

 As for the frequency of ocular symptoms, 63% of patients (n=123) reported experiencing complaints every month of the year. The main trigger for allergic symptoms, according to patients’ perception, as assessed through the trigger questionnaire, was house dust (87%), followed by changes in temperature (79%), pollution (67%) and contact with mold (54%), as illustrated in Table 2. 

**Table 2:** Apparent triggers of allergic crises from the patients’ perspective, assessed by trigger questionnaire.

Apparent trigger	Total n (%)	VKC/AKC n (%)	SAC/PAC n (%)	p-value
House dust	171 (87)	66 (89)	105 (86)	0.724
Temperature changes	155 (79)	55 (74)	100 (82)	0.099
Pollution	131 (67)	48 (65)	83 (68)	0.622
Mold	106 (54)	41 (55)	65 (53)	0.915
Respiratory infections	98 (50)	30 (41)	68 (56)	0.029*
Cats	87 (44)	35 (47)	52 (43)	0.590
Wind	84 (43)	36 (49)	48 (39)	0.238
Light/brightness	72 (37)	36 (49)	36 (30)	0.009*
Dogs	61 (31)	29 (39)	32 (26)	0.068
Pollens	44 (22)	16 (22)	28 (23)	0.782

Legend: VKC: vernal keratoconjunctivitis; AKC: atopic keratoconjunctivitis; SAC: seasonal allergic conjunctivitis; PAC: perennial allergic conjunctivitis.

 The median score on the EQ-5D visual analog scale was the same in both groups during the non-crisis period (100). During crises, participants with VKC/AKC had a significantly lower median score than those with SAC/PAC (30 [IQR 0–50] vs. 40 [IQR 20–60], respectively; p=0.04). The median total score on the QUICK quality of life questionnaire was the same in both groups, 26 (IQR 23–31 in the VKC/AKC group and IIQ 22–28 in SAC/PAC). The only question that showed a significant difference in responses between the groups was "During the last 2 weeks, because of conjunctivitis did you have to use eye drops?", with a significantly higher median score among patients with VKC/AKC (3 vs. 2; p=0.003). Table 3 presents median score on the QUICK questionnaire for each question and the median total score according to the diagnosis of ocular allergy. 

**Table 3:** Median score on the Quality of Life in Children with Keratoconjunctivitis questionnaire answered by children and adolescents with ocular allergy.

During the last 2 weeks, because of conjunctivitis...	VKC/AKC (n=74)	SAC/PAC (n=122)	p-value
Question 1: ... did you feel burning in your eyes?	1 (1–3)	2 (1–3)	0.253
Question 2: ... did you have trouble staying in an air-conditioned room?	1 (1–3)	1 (1–3)	0.938
Question 3: ... did you have to use tissues?	2 (1–3)	2 (1–3)	0.898
Question 4: ... did you have puffy eyes?	2 (1–3)	2 (1–3)	0.915
Question 5: ... did you have problems in the light?	2 (1–3)	1 (1–3)	0.084
Question 6: ... did you have tearing?	2 (1–3)	2 (1–3)	0.946
Question 7: ... did you have itchy eyes?	2 (1–3)	2 (1–3)	0.988
Question 8: ... did you have red eyes?	2 (1–3)	2 (1–3)	0.348
Question 9: ... did you have blurred vision?	2 (1–3)	1 (1–3)	0.097
Question 10: ... did you have eye secretions?	2 (1–3)	1 (1–3)	0.297
Question 11: ... did you have to use eye drops?	3 (1–3)	2 (1–3)	0.003*
Question 12: ... did you have closed and sticky eyes in the morning?	2 (1–3)	1 (1–3)	0.074
Question 13: ... did you have trouble playing outdoors?	1 (1–3)	1 (1–3)	0.363
Question 14: ... did you have trouble practicing sports (e.g., football and gym)?	1 (1–3)	1 (1–3)	0.795
Question 15: ... did you have trouble meeting your friends?	1 (1–3)	1 (1–3)	0.962
Question 16: ... did you have trouble going to the swimming pool?	1 (1–3)	1 (1–3)	0.872
Total score:	26 (16–40)	26 (16–43)	0.180

Legend: VKC: vernal keratoconjunctivitis; AKC: atopic keratoconjunctivitis; SAC: seasonal allergic conjunctivitis; PAC: perennial allergic conjunctivitis. 1=Never; 2=Sometimes; 3=Always.

Median (minimum and maximum).

**Figure 2:**
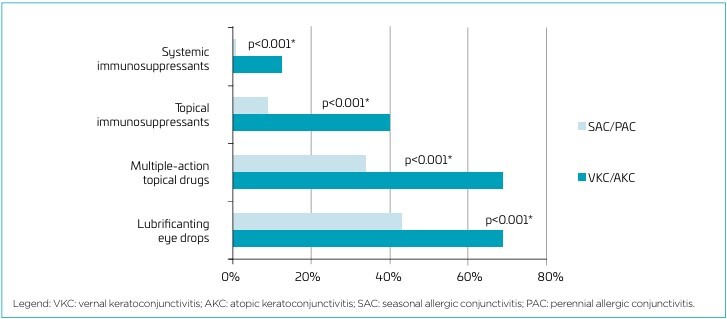
Frequencies of use of medication from different classes in patients with vernal keratoconjunctivitis and atopic keratoconjunctivitis and those with seasonal allergic conjunctivitis and perennial allergic conjunctivitis (n=187).

 For therapeutic control, 69% of patients with VKC/AKC were using lubricating eye drops and multiple-action topical drugs *versus* 43 and 34% of those with SAC/PAC (p<0.001). Topical immunosuppressants were used by 40% of patients with keratoconjunctivitis and 9% with allergic conjunctivitis (p<0.001), while systemic immunosuppressants were used in 13% of VKC/AKC and 0.8% of SAC/PAC patients, as illustrated in Figure 2. 

 When comparing the frequency of immunosuppressive drug use in patients diagnosed with VKC or AKC across the three centers participating in this study, higher rates were found in the coordinating center: 67.6% for topical immunosuppressants and 23.5% for systemic ones (UNIFESP) *versus* 19.2 and 3.8% (UFPR) and 14.3 and 7.1% (UFPE). 

## DISCUSSION

 The inclusion of centers from three distinct regions of the country (South, Southeast, and Northeast) aimed to ensure a broader and more representative sampling of patients with ocular allergy, while also accounting for potential regional variations in clinical presentation. In this study, there was a predominance of males (61.2%) among patients diagnosed with ocular allergy followed up in three reference centers for pediatric allergy in Brazil. Most previous studies have described a higher prevalence of ocular allergy symptoms in females, but more recent publications point to a trend towards a change in the prevalence of this disease between genders according to age group, with boys being more frequent during childhood and girls more frequent after puberty.^11,12^

 As observed by other researchers, there was a high frequency of association between the diagnosis of allergic conjunctivitis and other allergic comorbidities (rhinitis, asthma and atopic dermatitis). In a national study carried out in southern Brazil, it was estimated that 75% of patients with allergic rhinitis had eye symptoms, as did around 20% of patients with asthma.^4^

 Patients diagnosed with VKC and AKC — severe forms of ocular allergy observed in children and adolescents — were grouped together for analysis. Although VKC and AKC present distinct clinical features and prognoses, they share important similarities that differentiate them from SAC and PAC, particularly in terms of greater disease severity, increased need for pharmacological treatment, and a higher frequency of complications. SAC and PAC also exhibit substantial clinical similarity, which supported the decision to analyze these patient groups collectively. Furthermore, in Brazil, sensitization to seasonal aeroallergens such as pollens and grasses is relatively uncommon — except in certain areas of the Southern region — making it particularly challenging to distinguish between these subtypes of allergic conjunctivitis in clinical practice. 

 It is worth noting that the prevalence of the allergic comorbidities investigated did not differ between patients diagnosed with VKC or AKC compared to those with SAC or PAC, which corroborates the importance of the Th2 pathway in the pathogenesis of keratoconjunctivitis.^6^

 Regarding the intensity of ocular symptoms present in the last two weeks, pruritus and hyperemia stood out in both groups (VKC/AKC and SAC/PAC). Ocular pruritus is the most characteristic symptom of allergic conjunctivitis, and the diagnosis of this disease is unlikely in its absence.^13^ The only ocular symptom that differed between the groups was photophobia, which was more intense among patients with KC. Unexpectedly, ocular pain had a similar reported intensity between the patient groups, even though it is a symptom usually indicative of corneal involvement. We infer this result to a possible difficulty patients may have in interpreting symptoms such as itching, burning, or foreign body sensations and translating them into ocular pain. 

 As for the frequency of ocular symptoms throughout the year, the majority of patients (63%) reported having them every month. Although the literature indicates SAC as the most frequent form of ocular allergy, studies in tertiary referral centers show that the chronic forms are the most commonly seen by specialists. A study at a Brazilian ophthalmology center evaluated 207 patients with ocular allergy, 38% of whom were diagnosed with VKC, 39% with AKC, 13% with PAC and 10% of patients had no definite diagnosis.^14^ In addition, SAC is generally related to sensitization to pollens, which is described more frequently in regions with temperate climates. Perennial forms, on the other hand, are usually related to indoor allergens such as house dust mite, animal dander, cockroaches and fungal spores.^12^ As expected, the trigger that patients most frequently related to the onset of eye symptoms was house dust (mite) (87%). Strikingly, 79% of the participants pointed to variations in room temperature as a trigger for eye symptoms. As described in some studies, meteorological factors (humidity, temperature) can affect eye health directly, impacting the quantity and quality of the tear film, or indirectly, by raising circulating levels of allergens such as pollens.^15,16^

 Despite the potential negative impact on the visual capacity of patients, as well as repercussions on the quality of life and productivity of those affected, ocular allergy is still a disease that is often neglected and insufficiently treated in Brazil. A study carried out in southern Brazil showed that 43.9% of children and adolescents up to 14 years of age with asthma reported at least one ocular symptom, but only 15.8% received a medical diagnosis of allergic conjunctivitis, demonstrating that the disease is underestimated and underdiagnosed.^17^

 The use of two different instruments to assess the impact of ocular allergy on patients’ quality of life is justified by the differences in the scope of evaluation provided by each tool. The QUICK questionnaire directly addresses ocular symptoms and their effects on patients’ daily activities (e.g., photophobia, itching, limitation of outdoor activities, school-related impact), allowing for a detailed and sensitive analysis of the clinical and functional repercussions of the disease. In contrast, the EQ-5D Visual Analog Scale captures the patient’s overall perception of their current health status. It provides a broad, non-specific assessment that is useful for evaluating the general subjective perception of health, though it is less sensitive to specific ophthalmologic symptoms. The combined use of these two instruments enables a more comprehensive evaluation of quality of life, capturing both the specific impact of ocular allergy and the patient’s global perception of health. 

 In this study, the application of the EQ-5D visual analog scale showed a significant difference in patients’ general health status scores during and after an exacerbation of conjunctivitis. Notably, in the group of patients diagnosed with KC, the scores given during flares were significantly lower than in patients with seasonal and perennial allergic conjunctivitis, reflecting the greater clinical severity and functional impact associated with the more severe forms of ocular allergy. On the other hand, when evaluating the answers to the QUICK quality of life questionnaire, the same median total scores were found for both patient groups and the only item that demonstrated a statistically significant difference between the groups was related to the need for eye drop use, which was reported more frequently by patients with VKC/AKC, consistent with the known requirement for more intensive pharmacologic management in these cases. Though unexpected, this result suggests that SAC and PAC — despite typically presenting a milder clinical course with lower rates of ocular complications and less need for specific drug therapies — have a negative impact on patients’ quality of life comparable to that of KC. 

 The similarity in median total scores on the QUICK questionnaire across groups may also be justified by patients’ perception, where once symptoms interfere with daily life the referred impact on quality of life is similar even if the underlying condition is mild (SAC/PAC) or severe (VKC/AKC). Moreover, in the case of SAC and PAC, symptoms are generally milder, but may be persistent or recurrent, leading to cumulative impact on quality of life. On the other hand, patients with KC might underreport the impact of chronic symptoms over time. It is also possible that the questionnaire is not sufficiently sensitive to detect differences related to the intensity or chronicity of symptoms, as many items assess the presence of symptoms and their interference with daily activities in a categorical or ordinal manner, which could limit its discriminative power, especially in cases where both groups report the presence of symptoms. This finding highlights the importance of considering not only clinical severity but also patient-reported outcomes in the management of ocular allergy, since even less severe phenotypes demand appropriate follow-up and treatment strategies to improve quality of life. 

 Data from medical records regarding *in vivo/in vitro* allergic sensitization testing were not standardized, as they varied in terms of the time of collection and the method of investigation and were therefore not analyzed in the present study. It is important to note that allergic sensitization tests do not necessarily reflect the main symptom triggers, as they only assess allergic sensitization. Specific provocation tests would be required for this purpose, which were not feasible within the context of the study. 

 Regarding therapy, there was a higher prevalence of medication use across all the classes investigated among patients with VKC/AKC. This can be explained by the greater potential for severity and complications in these forms of ocular allergy. One striking finding is the low frequency of use of lubricating eye drops (43%) by patients with SAC/PAC, given that they are part of the recommendations for primary therapeutic intervention in the treatment of OA.^6^ The fact that this study was carried out in public health reference centers in Brazil may partly explain this finding, as these are medications that are not provided free of charge in the public network. Even so, the professionals responsible for these patients must remain vigilant to ensure that those with less severe clinical conditions receive the appropriate and necessary treatment according to their symptoms. 

 In conclusion, the clinical presentation of OA is heterogeneous among Brazilian children and adolescents, with pruritus and hyperemia being the most common symptoms in this sample. The concomitant occurrence of other allergic diseases is high, especially allergic rhinitis. The main trigger of ocular symptoms reported by patients and their families was house dust. Children and adolescents with KC showed a higher impact on quality of life during symptomatic periods than those with SAC and PAC. Ocular lubricants were underused by patients, especially in the milder forms of the disease (SAC/PAC). Individuals diagnosed with KC used medications from all the classes investigated in this study more frequently. 

## Data Availability

The database that originated the article is available with the corresponding author.
